# Self-ordered pointing and visual conditional associative learning tasks in drug-free schizophrenia spectrum disorder patients

**DOI:** 10.1186/1471-244X-8-6

**Published:** 2008-01-23

**Authors:** Emilio Sacchetti, Alessandro Galluzzo, Adelaide Panariello, Giovanni Parrinello, Stefano Francesco Cappa

**Affiliations:** 1 Department of Psychiatry, Brescia University School of Medicine, Brescia, Italy; 2 University Psychiatric Unit, Brescia University School of Medicine and Brescia Spedali Civili, Brescia, Italy; 3 Department of Mental Health, Brescia Spedali Civili, Brescia, Italy; 4 Center on Behavioural and Neurodegenerative Disorders, Brescia University and EULO, Brescia, Italy; 5 Department of Biomedical Sciences and Biotechnologies Section of Medical Statistics and Biometry, Brescia University School of Medicine, Brescia, Italy; 6 Departments of Psychology and Neuroscience, Vita e Salute San Raffaele University, Milan, Italy

## Abstract

**Background:**

There is evidence of a link between schizophrenia and a deficit of working memory, but this has been derived from tasks not specifically developed to probe working memory per se. Our aim was to investigate whether working memory deficits may be detected across different paradigms using the self-ordered pointing task (SOPT) and the visual conditional associative learning task (VCALT) in patients with schizophrenia spectrum disorders and healthy controls. The current literature suggests deficits in schizophrenia spectrum disorder patients versus healthy controls but these studies frequently involved small samples, broad diagnostic criteria, inclusion of patients on antipsychotic medications, and were not controlled for symptom domains, severity of the disorder, etc. To overcome some of these limitations, we investigated the self-monitoring and conditional associative learning abilities of a numerically representative sample of healthy controls and a group of non-deteriorated, drug-free patients hospitalized for a schizophrenia spectrum disorder with florid, mainly positive psychotic symptoms.

**Methods:**

Eighty-five patients with a schizophrenia spectrum disorder (DSM-IV-TR diagnosis of schizophrenia (*n *= 71) or schizophreniform disorder (*n *= 14)) and 80 healthy controls entered the study. The clinical picture was dominated by positive symptoms. The healthy control group had a negative personal and family history of schizophrenia or mood disorder and satisfied all the inclusion and exclusion criteria other than variables related to schizophrenia spectrum disorders.

**Results:**

Compared to controls, patients had worse performances on SOPT, VCALT and higher SOPT/VCALT ratios, not affected by demographic or clinical variables. ROC curves showed that SOPT, VCALT, and SOPT/VCALT ratio had good accuracy in discriminating patients from controls. The SOPT and VCALT scores were inter-correlated in controls but not in patients.

**Conclusion:**

The selection of a clinically homogeneous group of patients, controlled for a number of potential confounding factors, and the high level of significance found in the different analyses confirm the presence of SOPT and VCALT abnormalities in a large preponderance of patients with schizophrenia spectrum disorder with positive symptoms. SOPT, VCALT, and SOPT/VCALT ratio showed good accuracy in discriminating patients from healthy controls. These conclusions cannot be extended to schizophrenia spectrum disorder patients with a different clinical profile from our patient population.

## Background

A number of independent lines of evidence support the existence of a link between schizophrenia and a deficit of working memory. Many patients with schizophrenia share impairment of the processes responsible for the on-line maintenance of information necessary to select task-appropriate action [1,2]. Furthermore, working memory participates in many domains of functioning which are critical in schizophrenia [3,4]. The study of working memory is a valuable tool to investigate the prefrontal cortex [5], a brain region central to many leading hypotheses of schizophrenia [6].

Because most of the available evidence is derived from tasks not specifically developed to probe working memory per se, we wanted to investigate whether working memory deficits may be detected across different paradigms [1]. Within this perspective, a deeper understanding of the performance of schizophrenia patients on the self-ordered pointing task (SOPT) [7-9] and the visual conditional associative learning task (VCALT) [9-12] seems appropriate, given that these two tasks have been less extensively studied than others.

The SOPT, originally devised by Petrides and Milner [7], assesses the capacity to initiate and execute a sequence of responses with self-monitoring of performance and makes considerable demands on working memory as subjects must constantly compare responses already made with those remaining. Self-monitoring is considered an important aspect of executive function [13]. The VCALT [9,10] is based on the ability to learn associations between a set of stimuli and a set of responses; these fixed associations are acquired through a process of "trial and error learning", consisting of the ability to discriminate between past correct and incorrect responses and using this information to guide response selection. Some PET evidence suggests that the mid-dorsolateral frontal cortex, which comprises cytoarchitectonic Brodmann areas 46 and 9, may be associated with the performance of non-spatial self-ordered pointing tasks, while the adjacent area 8 of the posterior-dorsolateral frontal cortex may be activated during conditional associative learning tasks [9,14].

The current literature on SOPT [15-17] and VCALT [18-20] consistently supports the presence of deficits in schizophrenia patients versus healthy controls. However, these studies frequently involved small samples, utilized broad diagnostic criteria, included patients taking antipsychotic medications, and did not control for symptom domains, severity of the disorder and other putative sources of variation.

In an attempt to overcome some of these limitations, we investigated the self-monitoring and the conditional associative learning abilities of a numerically representative sample consisting of healthy controls and a group of non-deteriorated, drug-free patients hospitalized for a schizophrenia spectrum disorder with florid, mainly positive psychotic symptoms.

## Methods

### Subjects

All consenting males and females consecutively and voluntarily admitted over a 2-year period to the Brescia University and Spedali Civili Psychiatric Service were included, provided that they fulfilled predefined inclusion and exclusion criteria at the screening visit.

Inclusion criteria were a DSM-IV-TR diagnosis of schizophrenia, paranoid type, or of schizophreniform disorder [21] with prevalent delusional and/or hallucinatory symptoms, a minimum total score of 80 on the Positive and Negative Syndrome Scale (PANSS) [22], a score of 4 or more on at least two items of the PANSS positive subscale, a minimum antipsychotic drug-free interval of 4 weeks for oral formulations and 6 weeks for depot formulations, a Mini Mental State Examination (MMSE) [23] score higher than 24, a total intelligence quotient (IQ) score on the Wechsler Adult Intelligence Scale-Revised (WAIS-R) [24] higher than 75, and a level of understanding, cooperativeness and attention sufficient enough to perform neuropsychological tests and give informed consent after a detailed explanation of the procedures and the nature of the study. Exclusion criteria were older than 75 years, current diagnoses of DSM-IV-TR axis 1 or 2 co-morbid disorders, a history of substance-related disorders in the preceding 6 months, evidence of head trauma or seizures at any time during the lifespan, concomitant medical or neurological disorders presumptively able to affect the neuropsychological performance, and positive qualitative assays for alcohol and/or substance of abuse. Operatively, all participants underwent detailed clinical interviews implemented, when required, by a DSM-IV-TR adjusted version of the Structured Clinical Interview for DSM-IV Axis 1 Disorders, Clinician Version (SCID-CV) [25]. Predominance of positives symptoms was defined as a PANSS positive/negative subscale score ratio >1. The PANSS, the MMSE, and the WAIS-R were administered on the day following the screening visit and before starting on antipsychotic medication. Patients were also evaluated for age at the onset of schizophrenia. To establish this, the appearance of the first psychotic symptoms represented the pre-identified cut-off; in order to reach as accurate estimates as possible, direct information from patients was systematically retrieved along with data obtained from at least one close relative plus, when possible, previous medical reports.

The control group consisted of doctors, nurses, employees, and attendants of Brescia Spedali Civili, students of Brescia University, or their relatives. For recruitment, the controls had to be unrelated to other prospective participants, be unaware of the nature of the neuropsychological tasks involved in the study, have a negative personal and family history of schizophrenia and mood disorders, and satisfy all the inclusion and exclusion criteria applied to the patients, with the exception of those schizophrenia-related variables. All controls underwent detailed clinical interviews implemented, when required, by the Diagnostic Interview for Genetic Studies [26] for controls. To exclude a positive family history for psychosis and mood disorders, the controls and, in cases of doubt, their family members were interviewed following an ad hoc questionnaire, which incorporated in-depth information on first-degree relatives and more general data on second-degree relatives.

Patients and controls were also questioned about their school education.

A team of qualified psychiatrists were in charge of data collection, after training and assessment of inter-rater reliability. Reliability was estimated using videotapes played by actors in which 20 cases with a schizophrenia spectrum disorder or a mood disorder with psychotic features were represented (Cohen's Kappa = 0.80, *P *< 0.001 for schizophrenia diagnosis; Pearson's *r *= 0.91, *P *< 0.01 for PANSS score, Pearson's *r *= 0.92, *P *< 0.01 for age at disease onset).

DSM-IV-TR related variables were independently evaluated by two physicians and, in the case of discordance, a joint revision of the material took place in the presence of an independent referee who made the final decision after discussion. Rating scales and the remaining variables were rated by only one physician, on rotation.

All the procedures used in this study are an integral part of the routine employed at the Brescia University and Spedali Civili Psychiatric Unit. Therefore patients were requested to provide only a written informed consent for the utilization of data for research purposes, after they received an explanation about the aims of the study and the lack of economic interest. The invited partecipants had also an explicit guarantee of anonymity and the impossibility of identification datails as a unique number linked all the individual data.

### Sample key features

The sample consisted of 165 subjects of Italian ancestry: 71 schizophrenia patients, 14 schizophreniform disorder patients, and 80 healthy volunteers. Patients and controls did not differ from each other in age (36 ± 12 vs. 34 ± 10 years, *t *= 0.82, *P *= 0.411), sex distribution (49/36 vs. 37/43 male/female ratio, chi squared = 2.1, *P *= 0.411), and educational level (10.5 ± 3.9 vs. 10.1 ± 2.5 years of school, *t *= 0.22, *P *= 0.823). Patients and controls were also comparable as to the MMSE (29.11 ± 0.93 vs 29.29 ± 0.90, *t *= 1.27, *P *= 0.204) and the IQ (93.89 ± 4.95 vs 94.85 ± 4.71, *t *= 1.268, *P *= 0.207) scores.

Patients had a severe disorder (PANSS total score: mean 98, median 98, 95% CI 86–110) and showed wide inter-individual variability of both age at disease onset (years: mean 27.4, median 25, 95% CI 20–32) and duration of the disorder (years: mean 7.99, median 5, 95% CI 2–12). At the screening visit, 17 and 68 patients were drug-naive and drug-free, respectively. Among the drug-free patients, the last prescription involved second-generation antipsychotics in 85% of the cases.

### Neuropsychological tests

Once enrolled, patients and controls were assessed with both the SOPT and the VCALT. The SOPT and the VCALT do not have a pre-defined time limit; they are completed in about 7 and 13 min, respectively. To minimize possible influences on performance played by even subtle differences in the explanation of the two tasks, SOPT and VCALT were described using standardized written texts. The description of the two tests, the control and assistance during their completion, and the recording of the results were carried out by one of the psychiatrists responsible for the recruitment and the collection of all the data.

Briefly, the SOPT is based on four identical series of eight cards. On each card, the same eight black and white abstract designs are represented with a different, casual distribution. After explanation by the examiner on the type of performance required, the first card is disclosed. At this point, the candidate is invited to choose one of the eight designs. Then the second card is presented and the candidate is requested to select one of the seven remaining, unidentified designs. Thereafter, the candidate is invited to choose one design among those previously not selected whenever a new card is shown. The test ends after the same procedure is repeated for the second, third, and fourth card series. The SOPT score is given by the number of wrong answers, that is how many times the candidate selected, within each card series, a previously identified design.

The VCALT is based on eight identical series of four cards. The four cards of a series contain the same four black and white abstract designs but differ from each other in the internal distribution of the designs and the presence of a coloured strip (yellow, blue, green, or red) on the top. Before starting and after the explanation about how the test works, the examiner arbitrarily decides to associate any design with a specific colour. At this point the candidate, completely unaware of the four pre-established design-colour associations, uncovers the first card, links one of the four designs with the colour of the top strip, and receives from the examiner a communication whether the choice corresponds to the pre-established association or not. The same procedure is repeated with the second, the third, and the fourth card, respectively. Once the first card series is completed, the search for the same design-colour associations is renewed for the other seven card series, the initial choices of the examiner remaining valid. The final VCALT score is given by the number of correct answers, that is how many times the candidate correctly identified the pre-established design-colour associations.

During the two tests, the examiner registered the correct and incorrect answers without any comment about the performance.

In order to obtain some information about the independence or the interdependence of the two tasks, the SOPT and VCALT values were also to calculate a supplementary unitary index, the SOPT/VCALT ratio.

### Statistical analyses

Rough data, expressed as means (SD) or median (IQR) if continuous and as percentages if categorical, were analysed by the *t*-test or the chi-squared test. The possible confounding effects of key baseline characteristics on SOPT, VCALT and SOPT/VCALT values were studied by multivariate linear regression analyses. Logistic regression analyses were utilized to obtain the SOPT, VCALT and SOPT/VCALT adjusted odds ratios relative to schizophrenia spectrum disorder patients with respect to healthy controls. ROC analyses of the SOPT, VCALT and SOPT/VCALT values were performed in order to confirm the ability of the three neuropsychological measures to discriminate patients from controls and to estimate the best cut-offs, that is the values that minimize the absolute difference between sensitivity and specificity. The same ROC cut-offs valid for the VCALT, SOPT, and SOPT/VCALT ratio were then used to evaluate the positive and negative likelihood ratios which provide a direct estimate of how much the three neuropsychological values change the odds of having a schizophrenia spectrum disorder.

Inter-rater reliability was estimated using Cohen's kappa or Pearson's *r *according to the type of variable considered.

All the statistical analyses were performed using the 'R Language' freeware statistical package [27].

*P *values <0.05 were considered statistically significant.

## Results

**Figure 1:**
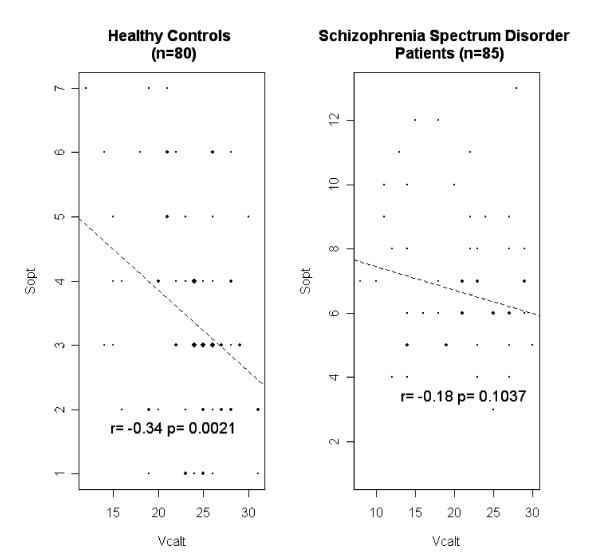
Correlations between the performances on SOPT and VCALT in healthy controls and schizophrenia spectrum disorder patients.

Patients differed remarkably from healthy controls in that they had more SOPT wrong answers, less VCALT right answers, and higher SOPT/VCALT ratios (Table 1). Patients with schizophrenia and those with schizophrenia spectrum disorder did not differ in SOPT, VCALT, and SOPT/VCALT values although a numerical advantage of patients with schizophreniform disorder emerged for the VCALT (Table 2). Furthermore, the SOPT and the VCALT scores were inter-correlated with each other in healthy controls but not in schizophrenia spectrum disorder patients (Fig. 1).

**Table 1:** Test performance for SOPT, VCALT and SOPT/VCALT ratio of schizophrenia spectrum disorder patients and healthy control subjects

	Schizophrenia spectrum disorders patients (*n *= 85)			Controls (*n *= 80)			Test statistics^a^	
	Mean (SD)	Median	25–75th percentile	Mean (SD)	Median	25–75th percentile	*t*	*p*

SOPT (wrong answers)	6.8 (2.4)	7.0	5.0–8.0	3.4 (1.6)	3	2.0–4.0	11.74	<0.001
VCALT (right answers)	18.5 (5.7)	18	14.5–23.0	23.5 (4.2)	24.0	21.0–26.0	6.40	<0.001
SOPT/VCALT ratio	0.42 (0.23)	0.364	0.25–0.50	0.15 (0.09)	0.125	0.09–0.20	12.32	<0.001

^a^Wilcoxon test.

**Table 2:** SOPT, VCALT and SOPT/VCALT ratio: comparison between schizophrenia and schizophreniform disorder patients

	Schizophrenia patients (71)			Schizophreniform disorder patients (14)			*F*_(1,83)_	*P*
	Mean ± SD	Median	25–75th percentile	Mean ± SD	Median	25–75th percentile		

SOPT	6.8 ± 2.3	7.00	5.0–8.0	7.1 ± 2.6	7.0	5.3–8.8	0.22	0.639
VCALT	17.8 ± 5.5	18	14.0–22.0	21.4 ± 6.5	22.0	16.5–26.3	3.80	0.055
SOPT/VCALT	0.43 ± 0.24	0.37	0.27–0.5	0.37 ± 0.19	0.3	0.25–0.45	1.10	0.292

^a^Wilcoxon test.

Multivariate linear regression analyses showed that SOPT, VCALT, and SOPT/VCALT values were not significantly affected by age, gender, and educational level in both the patient and the control groups and by duration of the disorder, age at the disease onset, and PANSS total score in the patient group (Table 3).

**Table 3:** Multivariate linear regression analyses of variables potentially affecting SOPT, VCALT and SOPT/VCALT values in patients and healthy controls

	**SOPT**			**VCALT**			**SOPT/VCALT**		
	Beta	SE	*P*	Beta	SE	*P*	Beta	SE	*P*

**Patients (*n *= 85)**									

Intercept	7.7415	1.956	0.0002	16.534	4.798	0.0009	0.54	0.195	0.0071
Age	0.0399	0.087	0.6485	0.107	0.214	0.6167	-0.0000	0.009	0.9982
Gender (f)	1.1765	0.627	0.0644	0.606	1.538	0.6945	0.039	0.062	0.5345
Educational level	-0.0443	0.066	0.5042	0.255	0.162	0.1188	-0.0089	0.007	0.1805
Duration of the disorder	-0.0282	0.087	0.7471	-0.227	0.214	0.2907	0.0046	0.009	0.5950
Age at onset	-0.0694	0.081	0.3952	-0.065	0.199	0.7441	-0.0032	0.008	0.6947
PANSS total	-0.0025	0.016	0.8763	-0.013	0.039	0.7395	0.0001	0.002	0.9463

**Controls (*n *= 80)**									

Intercept	1.6488	0.161	0.0000	4.7161	0.208	0.0000	0.3710	0.052	0.0000
Age	0.0041	0.003	0.1990	-0.003	0.004	0.4390	0.0011	0.001	0.2830
Gender (f)	0.0635	0.072	0.3840	0.0860	0.094	0.3620	0.0043	0.023	0.8540
Educational level	-0.0026	0.010	0.8060	0.0173	0.014	0.2110	-0.0032	0.003	0.3530
*Case vs control*	0.7756	0.0713	0.0000	-0.5809	0.0921	0.0000	0.2479	0.023	0.0000

Logistic regression analyses demonstrated that SOPT, VCALT, and SOPT/VCALT adjusted for age, gender, and educational level discriminate schizophrenia spectrum disorder patients from controls (Table 4).

**Table 4:** Logistic regression including the performances on SOPT, VCALT, age and educational level

	Odds-ratio^a^	Lower	Upper	*P*
SOPT	39.63	13	120	<0.001
VCALT	0.18	0.09	0.34	<0.001
SOPT/VCALT	35.91	12	108	<0.001

^a^Adjusted for age, gender and educational level.

**Figure 2:**
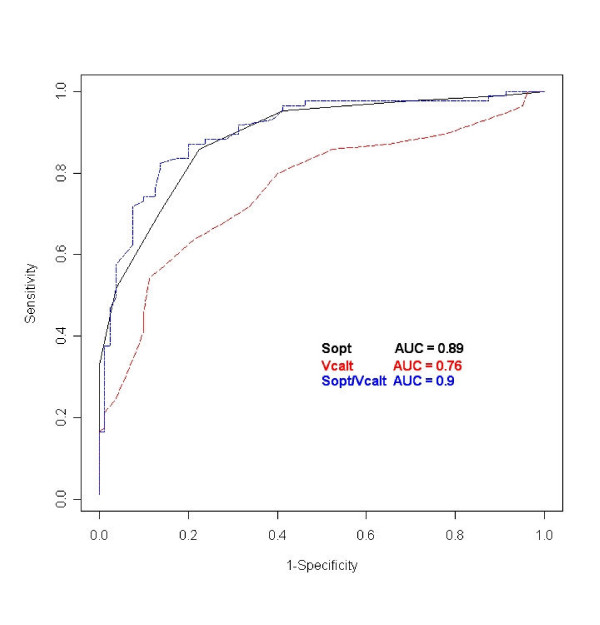
Areas under the ROC curves of SOPT, VCALT and SOPT/VCALT ratio.

The ROC analyses confirmed a valuable patient-control separation as the SOPT, VCALT, and SOPT/VCALT areas under the curve exceeded 0.5 (Fig. 2), and demonstrated that a SOPT score >5, a VCALT score <21, and a SOPT/VCALT ratio >0.24 gave the best discriminatory accuracy (Fig. 2 and Table 5). Moreover, ROC positive and negative likelihood ratios for the three neuropsychological measures indicated numerically lower, though not statistically significant, odds for a schizophrenia spectrum disorder utilizing the VCALT rather than the SOPT or the SOPT/VCALT ratio cut-off (Table 6).

**Table 5:** ROC areas under the curves (AUC) and cut-off values

	AUC	SE	*z *value	Lower 0.95	Upper 0.95	Cut-offs^a^
SOPT	0.89	0.025	15.7	0.84	0.94	5.00
VCALT	0.76	0.038	6.8	0.69	0.83	21.00
SOPT/VCALT	0.90	0.025	16.3	0.85	0.95	0.24

^a^"Abnormal" performance: SOPT ≥ 5; VCALT ≤ 21; SOPT/VCALT ≥ 0.24.

**Table 6:** ROC positive and negative likelihood ratios^a^

	Positive likelihood	Lower 0.95	Upper 0.95	Negative likelihood	Lower 0.95	Upper 0.95
SOPT	5.1	3.0	10.3	0.34	0.23	0.47
VCALT	3.6	2.2	6.6	0.49	0.36	0.65
SOPT/VCALT	6.0	3.6	11.4	0.20	0.12	0.32

^a^The likelihood ratio incorporates both the sensitivity and specificity of the test and provides a direct estimate of how much a test result will change the odds of having a disease.

## Discussion and conclusion

Three key points best summarize the results of our study. First, acute patients with a schizophrenia spectrum disorder performed worse in SOPT and VCALT and had higher SOPT/VCALT ratios versus a group of healthy controls matched for age, sex, and educational level. Second, the cut-offs derived from the ROC curves showed that SOPT, VCALT, and SOPT/VCALT ratio had good accuracy in discriminating patients from controls. Third, the SOPT and the VCALT performances were inter-correlated with each other in the healthy controls but not in the patient group.

These results are not likely due to chance. Indeed, the sample of patients and controls was sizeable; the selection criteria limited the recruitment a priori to a rather clinically homogeneous group of patients, and a discrete number of possible demographic and clinical confounders had been statistically controlled. Given that important limitations inherent in many pre-existing studies on the same tasks [15-20] have been bypassed, the conclusion that a large preponderance of intellectually normal, non-deteriorated, antipsychotic drug-free schizophrenia spectrum disorder patients with a florid phase of psychosis dominated by positive symptoms have evident SOPT and VCALT deficits seems therefore fully supported.

However, the adoption of stringent criteria for the selection of the patients is both a strength and a weakness of the study because it prevents generalization of the results to schizophrenia spectrum disorder patients with different clinical features and/or currently exposed to antipsychotic medications.

Within these definite boundaries, some further comments can be made. As far as the timing of appearance of SOPT and VCALT abnormalities is concerned, the lack of any correlation with the duration of the disorder in a sample of patients that included a significant minority of subjects with schizophreniform disorder suggests that the observed defects are likely to be phenotypic expressions of underlying processes which have been structured and operant at least since the beginning of the illness. However, this conclusion does not exclude the possibility that, in the long-term, the progress of the psychosis and/or the inevitable associated pharmacological manipulation could exert supplementary effects on the two tasks. The observed trend for a worse VCALT performance of schizophrenia patients versus those with a schizophreniform disorder could fit with this last possibility.

The result that SOPT, VCALT, and SOPT/VCALT ratio abnormalities were frequently but not systematically associated in patients with otherwise largely common clinical features could be compatible with the hypothesis that two tasks are sustained by only partially coincident mechanisms.

The ROC likelihood ratios suggest more frequent dysfunction of the SOPT and agree with the proposal [28,29] that deficits in self-monitoring play a principal role when positive symptoms dominate the clinical picture.

Since self-monitoring ability and conditional associative learning also involve the participation of other relevant cognitive processes such as attention, active memory, and inhibition, it seems acceptable to place our results within the general scenario of the "context processing" theory of cognitive dysfunctions of schizophrenia [30-33]. The involvement of multiple cognitive processes in the generation of SOPT and VCALT performance makes the search for a restricted anatomic specificity of the two tasks overtly hard and naive, even though neuroimaging [9,34] and lesion [7,10,12] studies suggest different roles for the Brodmann areas 46 and 9 and Brodmann area 8 in the spatial self-ordered pointing tasks and conditional associative learning, respectively.

The loss of correlation between SOPT and VCALT moving from healthy controls to patients may infer that a structural and/or functional disruption of the normally cooperative action between the networks involved should also exist in schizophrenia spectrum disorders. Within this proposal, a relative redundancy of the impairment of one network with respect to the other and/or a pathology of circuits crucial for their connection could be advocated. While we wait for direct investigations with neuroimaging techniques and multiple neuropsychological tasks, it is of interest to remember that functional disconnectivity has been proposed as crucial in the pathogenesis of schizophrenia [35] and that abnormal cortical functional connectivity has been demonstrated during impaired working-memory tasks [36-42].

In conclusion, further research seems warranted in order to firmly establish how much the SOPT and the VCALT paradigms may represent a valid tool to evaluate working memory in patients with schizophrenia or related disorders. The issues that merit priority are: whether and to what degree the individual SOPT, VCALT, and SOPT/VCALT values are stable over time, are affected by various treatments with antipsychotics, and are influenced by symptom clusters outside those of the positive domain.

## Competing interests

The author(s) declare that they have no competing interests.

## Authors' contributions

ES and AG participated in all aspects of the study. AP administered the tests to the patients. GP participated in the statistical analysis. SFC participated in the initial design of the study.

All authors have read and approved the final version of the manuscript.

## Pre-publication history

The pre-publication history for this paper can be accessed here:


